# Cholesterol-25-hydroxylase Is a Chicken ISG That Restricts ALV-J Infection by Producing 25-hydroxycholesterol

**DOI:** 10.3390/v11060498

**Published:** 2019-05-30

**Authors:** Tingting Xie, Min Feng, Manman Dai, Guodong Mo, Zhuohao Ruan, Guiyan Wang, Meiqing Shi, Xiquan Zhang

**Affiliations:** 1 Guangdong Provincial Key Laboratory of Agro-animal Genomics and Molecular Breeding, College of Animal Science, South China Agricultural University, Guangzhou 510642, China; ttxiehnnydx@126.com (T.X.); mgd9527@163.com (G.M.); zhuohaoruan@163.com (Z.R.); 18814116204@163.com (G.W.); 2 Key Lab of Chicken Genetics, Breeding and Reproduction, Ministry of Agriculture, Guangzhou 510642, China; 3 College of Veterinary Medicine, South China Agricultural University, Guangzhou 510642, China; daimanman1229@163.com; 4 Division of Immunology, Virginia-Maryland Regional College of Veterinary Medicine, University of Maryland, College Park, MA 20742, USA; mshi@umd.edu

**Keywords:** chicken, ISG, ALV-J, *CH25H*

## Abstract

The avian leukosis virus subgroup J (ALV-J) belongs to the chicken retrovirus that causes enormous economic losses in the poultry industry. Interferon-stimulated genes (ISGs) are critical for controlling virus infections. Here, we identified 897 type I ISGs induced by interferon-α (IFN-α) in chicken peripheral blood mononuclear cell (PBMC) by RNA-Seq. In addition, we further identified 152 potential anti-ALV-J chicken type I ISGs. Among these potential anti-ALV-J ISGs, chicken cholesterol 25-hydroxylase (*chCH25H*) was selected for further antiviral mechanism studies in chicken embryo fibroblast cell lines (DF1). The gene *chCH25H* is located on chromosome 6 and clustered in a distinct group with mammals *CH25H* in the phylogenetic tree. The core promoter region of *chCH25H* was located within −75/−1 sequence. We found that *chCH25H* was induced by chicken IFN-α and ALV-J in DF1 cells. The overexpression of *chCH25H* significantly inhibited ALV-J replication in DF1 cells at 48 h post infection (hpi). In addition, ALV-J replication was significantly enhanced in the chCH25H- knockout DF1 cells. Furthermore, we demonstrated that *chCH25H* restricted ALV-J infection through the production of 25-hydroxycholesterol (25HC), rather than type I and II interferon. Our results identified 152 potential anti-ALV-J chicken type I ISGs and revealed that 25HC, the product of *chCH25H*, could be used as a natural antiviral agent to control ALV-J infection.

## 1. Introduction

The avian leukosis virus subgroup J (ALV-J) is a member of the a-retrovirus genus of retroviridae, causing tumor disease, immunosuppression, and other secondary diseases in chicken. ALV-J has caused enormous losses to the world poultry industry at the end of the last century [1]. The prevention and control of ALV-J is mainly through eliminating infected chickens due to lacking effective vaccines and drugs. The western world has been successful in the eradication of ALV-J from the breeding flocks. However, the ALV purification of chicken flocks is a big project that requires a lot of manpower, materials and technology. Especially in developing countries like China, where farms lack management experience and sufficient funds, coupled with the existence of numerous local chicken breeds, ALV purification is a challenge. Therefore, it is important to change the individual’s resistance to ALV-J from the nature of the genetics, although it is difficult. The screening and identification of anti-ALV-J genes is an important strategy in the genetic improvement of chickens. However, to our knowledge, the systematic identification of anti-ALV-J genes has not been reported.

Stimulation with interferon (IFN) induces the expression of hundreds of genes defined as IFN-stimulated genes (ISGs) [2,3]. Hundreds of human ISGs have been identified, and some of them have been clarified for their antiviral functions [4,5,6]. However, the functions of the majority of antiviral ISGs remain unknown [3]. In addition, the functional mechanism of each ISG is various, resulting in greater selection difficulty of specific ISG research. In chickens, IFN-α induced ISGs have been also identified in primary embryo fibroblasts [7]. However, chicken ISGs that have been identified to have antiviral properties are still rare.

In the present study, taking ISGs as a starting point, we systematically identified potential anti-ALV-J ISGs and elucidated the antiviral molecular mechanism of chicken cholesterol 25-hydroxylase (*chCH25H*). Previously, it has been reported that *CH25H* broadly inhibited viral entry by production of 25-Hydroxycholesterol (25HC) [8]. As a soluble oxysterol, 25HC could inhibit viral entry by blocking membrane fusion between the cell and virus [8]. In addition, 25HC also acts as an amplifier of inflammation via AP-1 and that the resulting alteration in inflammatory response leads to increased tissue damage in mice following influenza infection [9], and acts as a modulator for both innate and adaptive immune responses toward inhibiting simian immunodeficiency virus (SIV) infection [10].

## 2. Materials and Methods

### 2.1. Ethics Statement

Chickens are used only for blood collection in this study. All animal research protocols were approved by South China Agriculture University’s Institutional Animal Care and Use Committee. (Permit Number: 2017003).

### 2.2. Cells and Virus

Specific-pathogen-free (SPF) White Leghorn chickens, half females and half males, were purchased from Guangdong DaHuaNong Animal Health Products Co., Ltd. (Guangzhou, China) and housed under pathogen free conditions. Peripheral blood mononuclear cell (PBMC) were isolated from blood aseptically collected from SPF chickens using chicken lymphocyte separation medium (Solarbio, Beijing, China). Chicken embryo fibroblast cell lines, DF1 cells, are known to be susceptible only to exogenous ALV [11] and were obtained from ATCC (Manassas, VA, USA). The ALV-J strain SCAU-HN06 was kindly provided by Prof. Ming Liao, South China Agricultural University. Infection with ALV-J strain SCAU-HN06 leads to haemangioma development.

### 2.3. Chicken PBMC Were Treated with ALV-J and IFN-α

PBMC were immediately infected with ALV-J strain SCAU-HN06 (10^4^ TCID_50_/0.1 mL) and treated with chicken IFN-α (chIFN-α) (1000 U/mL, GenWay Biotech, San Diego, CA, USA) for 6 h, respectively. Normal PBMC were used as a normal control (NC). PBMCs could respond to IFN [12], and chicken PBMCs could be infected with ALV-J [13]. Total RNA for RNA sequencing (RNA-seq) was isolated from these samples using TRIzol reagent (Invitrogen, Carlsbad, CA, USA). 

### 2.4. Library Preparation for mRNA Dequencing

Samples collected from two independent experiments were used for RNA-seq. After total RNA was extracted, mRNA was enriched by Oligo(dT) beads. Then the enriched mRNA was fragmented into short fragments using fragmentation buffer and reversely transcripted into cDNA with random primers. Second-strand cDNA were synthesized by DNA polymerase I, RNase H, dNTP and buffer. Then the cDNA fragments were purified with QiaQuick PCR extraction kit, end repaired, poly(A) added, and ligated to Illumina sequencing adapters. The ligation products were size selected by agarose gel electrophoresis, PCR amplified, and sequenced using Illumina HiSeq^TM^ 2500 by Gene Denovo Biotechnology Co. (Guangzhou, China). The sequencing data were deposited in the Bioproject database under the Bioproject ID: PRJNA430736, SRA ID: SRP130713.

### 2.5. Bioinformatic Analysis of RNA-Seq Data

To acquire high quality clean reads, the raw reads were filtered by removing the adapter-containing reads, low-quality bases including reads with more than 10% unknown nucleotides and low-quality reads with more than 50% of low quality bases (*Q*-value ≤ 20). The short reads alignment tool Bowtie2 was used for removing ribosomal RNA (rRNA) [14]. The remaining reads were mapped to the chicken genome assembly (Gallus_gallus-5.0) using TopHat2 (version 2.0.3.12) [15]. The mapped reads of each sample were assembled by software Cufflinks and Cuffmerge. Then, gene abundances were quantified by software RSEM [16] and the gene expression level was normalized by FPKM (Fragments Per Kilobase of transcript per Million mapped reads). 

In order to identify differentially expressed genes (DEGs) across groups (ALV-J vs NC, IFN-α vs NC), the edgeR package was used. Genes with fold change of |log_2_^FC^| > 1 (FC: fold change) and a false discovery rate (FDR) < 0.05 was considered as DEGs. 

### 2.6. Identification of Type I ISGs with Anti-ALV-J Potential

Compared to the NC group, the up-regulated DEGs induced by chIFN-α were identified as chicken type I ISGs in the present study. Then, DEGs induced by ALV-J were intersected with type I ISGs using software Venny-2.1. In this intersection, up-regulated DEGs induced by ALV-J were regarded to have the potential for anti-ALV-J activity.

### 2.7. Validation of chCH25H Expression in DF1 Cells

Recent studies have found that *CH25H* broadly inhibited infection by several viruses such as human immunodeficiency virus (HIV), Ebola virus, Zika virus, Porcine reproductive and respiratory syndrome virus, porcine intestinal coronavirus, mammalian reovirus, SIV and some fish viruses [8,10,17,18,19,20,21]. Accordingly, *chCH25H* was selected to further explore its anti-ALV-J function and mechanism in DF1 cells. Compared with PBMC, DF1 is easy to culture and is widely used in the validation test of chicken related research in vitro. To demonstrate whether *chCH25H* is induced by chIFN-α and ALV-J infection, DF1 cells were treated with chIFN-α (1000 U/mL) and ALV-J (10^4^ TCID_50_/0.1 mL). After 24 h and 48 h, *chCH25H* expression was measured by real-time quantitative PCR (qPCR) with published primers [22].

### 2.8. Chromosomal Location and Phylogenetic Analysis of chCH25H

To identify the characteristics of *chCH25H*, major species including human, mouse, dog, pig, horse, duck, monkey and chicken were collected from the National Center for Biotechnology Information (NCBI). The relative loci around *CH25H* genes in chicken and compared species were showed on simulated chromosome map. Phylogenetic analysis was performed using MEGA6. The NCBI accession numbers of *CH25H* are summarized as follows: human (NM_003956.3), mouse (NM_009890.1), dog (NM_001313827.1), pig (XM_005671263.3), horse (XM_001503007.4), duck (XM_005029425.3), monkey (NM_001198699.1), and chicken (NM_001277354.1).

### 2.9. Promoter Region Analysis of chCH25H

To identify the promoter region of *chCH25H*, the 2077 bp nucleotide sequences before the *CH25H* coding region were truncated into 6 segments to construct different dual luciferase reporter vectors. The first nucleotide before the *chCH25H* coding region is numbered −1, and the different dual luciferase reporter vectors are named PGL3- p2077(−2077/−1), p1000 (−1000/−1), p500 (−500/−1), p250 (−250/−1), p125 (−125/−1) and p75 (−75/−1), respectively (Figure 4A). The primers used to construct PGL3 plasmids were showed in Supplementary Table S1.

The PGL3 plasmids (p2077, p1000, p500, p250, p125, p75) were co-transfected with pRL-TK in DF1 cells, respectively. The pGL3-basic was co-transfected with pRL-TK as a control. Firefly and Renilla luciferase activities were measured at 24 h post-transfection using a Dual-GLO Luciferase Assay System Kit (Promega, Madison, WI, USA). Luminescence was measured using a Fluorescence/Multi-Detection Microplate Reader (BioTek, Shoreline, WA, USA). Firefly luciferase activities were normalized to Renilla luminescence in each well.

Furthermore, the common interferon stimulated response elements (ISRE) consensus 5’ A/GGTTTCN(1–2)TTTCC/T3’ or its reverse complement, and the common gamma activated sequence (GAS) consensus 5’ TTNCNNNAA3’ were screened at upstream of *chCH25H* according to previous studies [23].

### 2.10. Overexpression of chCH25H and Measurement of ALV-J Replication in DF-1cells 

The overexpression plasmid of *chCH25H* was constructed by cloning the *chCH25H* coding sequence from chicken PBMC into the pCMV-flag vector (Full name: CMV-SP6-3 × flag-CmR-ccdb-SV40 pA; SIDANSAI, Shanghai, China). DF1 cells were transfected with *chCH25H* plasmid by Lipofectamine 3000 reagent according to the manufacturer’s instructions (Invitrogen, USA). pCMV-EGFP was used as the a control. At 24 h post-transfection, transfected DF1 cells were infected with ALV-J strain SCAU-HN06 (10^4^ TCID_50_/0.1 mL). RNA, cell supernatants and total protein samples were collected at 24 and 48 hpi to detect the replication of ALV-J.

### 2.11. Construction of chCH25H Knockout DF1 Cell Line Using CRISPR/Cas9

The online design software (http://crispr.mit.edu/) was employed to design the gRNA of *chCH25H* based on the protospacer adjacent motif (NGG). The gRNA sequences of *chCH25H* were CTGCACAATGGAGAGACAGC (TGG). Target-sense sequences: 5’-CTCTTAGTCCTGCACAATGGAGAGACAGC-3’; Target-anti sequences: 5’-CTCTAAAACGCTGTCTCTCCATTGTGCAG-3’ (the sequences CTCTTAGTC and CTCTAAAAC were restriction sites). A poultry Cas9/gRNA Kit (Viewsolid Biotech, Beijing, China) was used to knockout the *chCH25H* in DF1 cells [24].

DF1 cells were transfected with the Cas9/gRNA recombinant plasmid of *chCH25H* and were screened with 2.5 μg/mL puromycin in DMEM with 10% FBS. Green fluorescence was observed by fluorescence microscope (Nikon, Japan) to show the proliferation of transfected-positive DF1 cells for 18 days. Total DNA was extract from these cells and was used as template to amplify *chCH25H* coding sequences. These *chCH25H* coding sequences were cloned into pMD-18T vector (Takara, Japan) and were transformed into DH5α (TransGen Biotech, Beijing, China). Twenty monoclonal bacteria were randomly selected for sequencing to measure the knockout efficiency.

The expression of *chCH25H* in *chCH25H* knockout cell line was detected by qPCR. These *chCH25H* knockout cells were infected with ALV-J strain SCAU-HN06. RNA samples were collected at 24 and 48 hpi to detect ALV-J replication.

### 2.12. 25HC Treatment

In order to determine the optimal concentration of 25HC, DF1 cells were treated with 25HC (Sigma Aldrich, Louis, MO, USA) at concentrations of 0.25, 0.5, 1, 2, and 4 μM for 24 h. Cytotoxicity was determined using Cell Counting Kit-8 dye (TransGen Biotech, Beijing, China). Subsequently, DF1 cells were pretreated with 25HC (optimum concentration) for 12 h. DF1 cells pretreated with same dose of ethanol were used as control. These DF1 cells were infected with SCAU-HN06 strain. qPCR and ELISA were used to determine the effect of 25HC on ALV-J replication at 24 hpi and 48 hpi. The same treatment is repeated in *chCH25H* knockout DF1 cell line.

In order to simulate the process of *CH25H* exerting antiviral function through 25HC, cell supernatants were collected from DF1 cells transfected with *CH25H* at 24 h post transfection and were used to treat other DF1 cells. Cell supernatants collected from DF1 cells transfected with empty vector were uesed as control. The methods of ALV-J infection are the same as the related experiments of 25HC above.

### 2.13. mRNA Quantification

A qPCR analysis was performed on a Bio-Rad CFX96 Real-Time Detection System using iTaqTM Universal SYBR^®^ Green Supermix Kit reagents (Bio-Rad, Hercules, CA, USA) according to the manufacturer’s specifications. The synthesis of cDNA was performed using a PrimeScript RT Reagent Kit (TaKaRa, Kusatsu, Japan) according to the manufacturer’s protocol. qPCR was employed to detecting the replication of ALV-J in mRNA level with the specific primers of ALV-J *gp85* gene [25]. The GAPDH gene was used as an internal control. Data analyses were performed using the 2^−ΔΔCt^ method.

### 2.14. Enzyme-Linked Immunosorbent Sssay (ELISA) 

The cell supernatants were tested for ALV group-specific antigen (p27) using the Avian Leukosis Virus Antigen Test Kit (Zoetis, Brooklyn, NY, USA) according to the manufacturer’s instructions. The results were expressed as s/p ratios where s/p = (Sample Mean–Kit Negative Control Mean)/(Kit Positive Control Mean–Kit Negative Control Mean). 

ELISA for chicken IFN-α, IFN-β and IFN-γ determination were obtained from Shanghai Enzyme-Linked Biotechnology Co. Ltd. (Shanghai, China). The ELISA experiments were performed according to the manufacturer’s specifications.

### 2.15. Western Blot Analysis

Western blotting was performed with ALV-J envelope protein specific mouse anti-monoclonal antibody JE9 (kindly provided by Prof. Aijian Qin, Yangzhou University, Yangzhou, China), rabbit anti-β-actin antibody (Bioworld, Louis Park, MN, USA) and mouse anti-flag monoclonal antibody (Proteintech, Rosemont, CA, USA) according to our previously described method [26]. IRDye 800-conjugated anti-mouse IgG and IRDye 700DX-conjugated anti-rabbit IgG (Rockland Immunochemicals, Limerick, PA, PA, USA) were used as the secondary antibody. Results were visualized and analyzed with an Odyssey FC infrared imaging system (LICOR Biosciences, Lincoln, NE, USA).

### 2.16. Statistical Analyses

All experiments were performed independently at least two times and each experiment was performed in triplicate. Statistical comparisons were performed using GraphPad Prism 5 (GraphPad Software Inc., San Diego, CA, USA). Results are presented as means ± SEM, and statistical significance was assessed at *P* values of < 0.05, 0.01, or 0.001.

## 3. Results

### 3.1. Identification of Potential Anti-ALV-J ISGs

**Figure 1:**
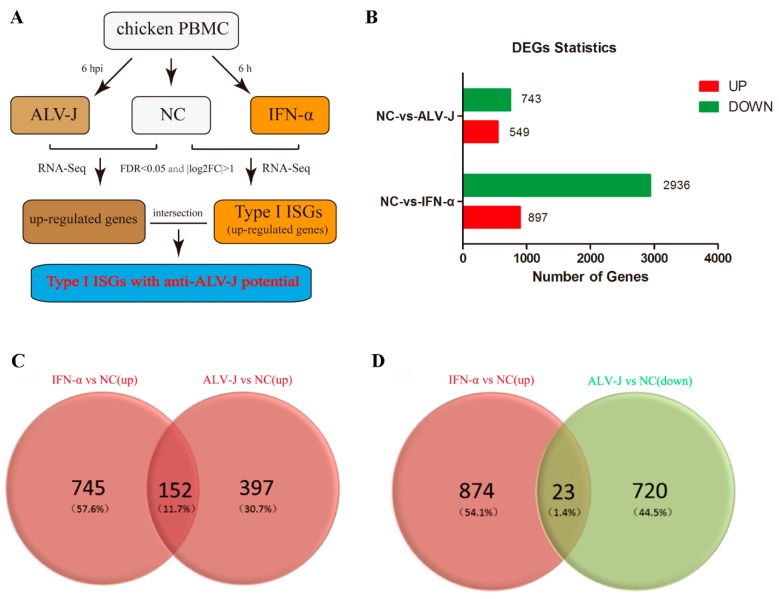
Identification of potential anti-ALV-J ISG candidates. (**A**) Summary of the strategy used to identify chicken type I ISGs and anti-ALV-J ISG candidates. (**B**) DEGs in PBMC cells infected with ALV-J, and DEGs in PBMC treated with chicken IFN-α. (**C**) Venn diagrams of up-regulated DEGs in the PBMC cells infected with ALV-J and treated with chicken IFN-α. DEGs in the intersection are considered as anti-ALV-J ISG candidates. (**D**) Venn diagrams of up-regulated DEGs in the PBMC cells treated with chicken IFN-α and down-regulated DEGs in the PBMC cells infected with ALV-J.

Two pipelines were employed for mining potential anti-ALV-J ISGs induced by type I IFN (Figure 1A). Chicken PBMC were treated by ALV-J and IFN-α for 6 h, respectively. Compared to untreated PBMC, DEGs were identified from ALV-J-infected group and IFN-α-treated group by RNA-Seq. Up-regulated DEGs induced by IFN-α were considered as chicken type I ISGs. The intersection of chicken type I ISGs and up-regulated expression DEGs induced by ALV-J were considered as potential anti-ALV-J ISGs (Figure 1A).

The Illumina HiSeq 2500 platform produced 164,635,966 raw reads. After discarding adaptor and low-quality sequences, we obtained 153,907,078 clean reads. The clean reads were mapped onto the chicken reference genome (Gallus_gallus-5.0), and the mapping rate of each library ranged from 75.58–77.45% (Supplementary Table S2). 

Compared to normal PBMC, 549 and 897 DEGs were significantly up-regulated in ALV-J-infected PBMC and IFN-α-treated PBMC, respectively (Figure 1B). 152 up-regulated DEGs were identical in ALV-J-infected PBMC and IFN-α-treated PBMC (Figure 1C). These 152 up-regulated PBMC were considered to be potential anti-ALV-J ISG candidates (Supplementary Table S3). In addition, 23 DEGs were up-regulated in IFN-α-treated PBMC cells but down-regulated in ALV-J-infected PBMC (Figure 1D). More details of these DEGs were showed in Supplementary Table S3.

### 3.2. The Expression of chCH25H Can Be Induced by ALV-J and IFN-α in DF1 Cells

**Figure 2:**
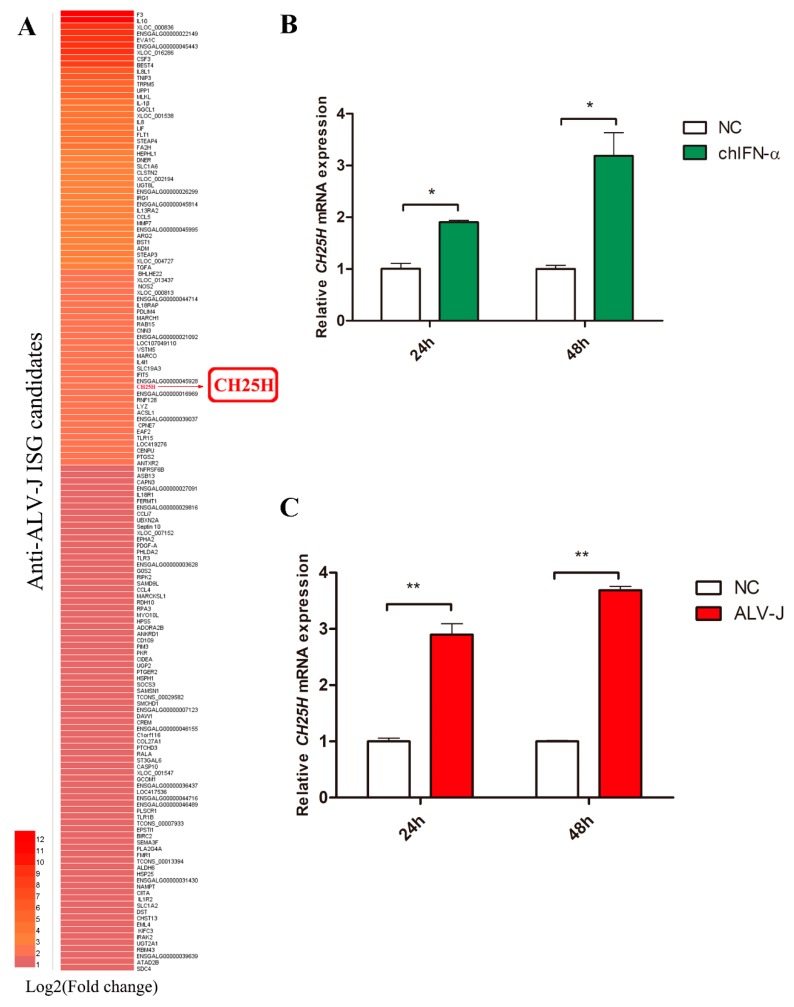
The expression of *chCH25H* is upregulated by ALV-J infection and IFN-α treatment in DF1 cells. (**A**) Heatmap of 152 chicken anti-ALV-J ISG candidates. (**B**) The expression of *chCH25H* was significantly increased in DF1 cells after treatment with 1000 U/mL IFN-α 24 h and 48 h. (**C**) DF1 cells were infected with ALV-J strain SCAU-HN06. *chCH25H* expression was significantly increased at 24 and 48 hpi. * *p* < 0.05; ** *p* < 0.01.

A heat map of 152 potential anti-ALV-J ISGs showed that some well-characterized antiviral ISGs such as *IFIT5*, *PKR* and *CH25H* were significantly up-regulated by chIFN-α and ALV-J (Figure 2A). Given that the broad antiviral effects of *CH25H* [8,10,17,18,19,20,21], *chCH25H* was selected to further explore its anti-ALV-J function and mechanism in DF1 cells.

DF1 cells were treated with IFN-α and SCAU-HN06, respectively. Gene expression analysis by qPCR showed that *chCH25H* is induced by both chIFN-α treatment and ALV-J infection in DF1 cells at 24 hpi and 48 hpi (Figure 2B,C). This result proved that *chCH25H* is a type I ISG, and indicated that it might play important role in anti-ALV-J. 

### 3.3. Genomic Architecture and Phylogenetic Analysis of chCH25H

**Figure 3:**
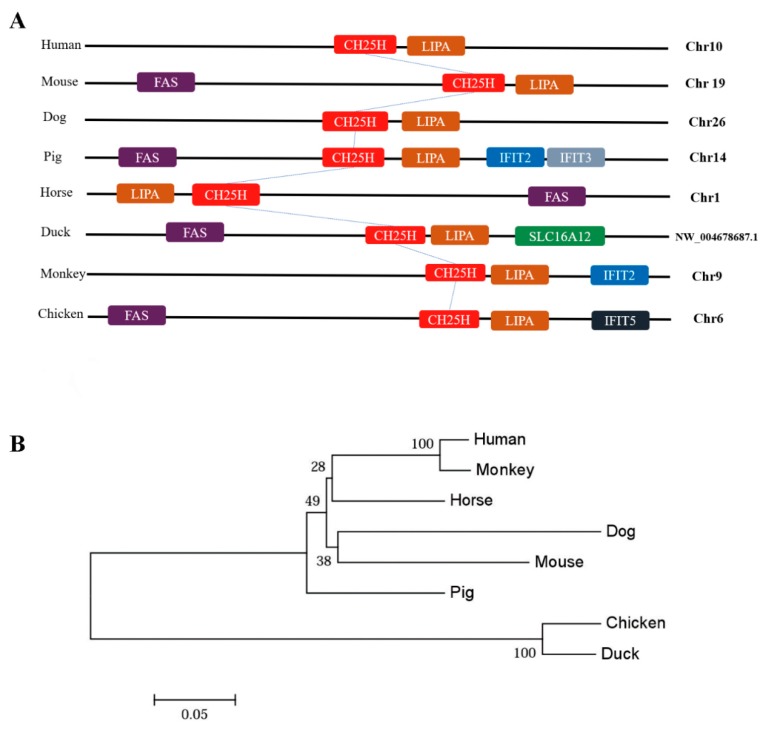
Chromosomal location and phylogenetic analysis of *chCH25H.* (**A**) Genomic architecture of *CH25H* genes in human, mouse, dog, pig, horse, monkey, duck and chicken. All of the *CH25H* locus in compared species is flanked with LIPA gene. *chCH25H* located on chicken chromosome 6. (**B**) Phylogenetic analysis of *CH25H* genes in different species. The chicken and duck *CH25H* genes clustered together in a group distinct from those of mammals.

The results showed that *CH25H* of each species were located on different chromosomes, and only the *chCH25H* located on chromosome 6 (Figure 3A). Interestingly, the *CH25H* gene of all species we compared was adjacent to the lysosomal acid lipase/cholesteryl ester hydrolase (*LIPA*) gene (Figure 3A). A previous study has shown that vertebrate *LIPA* and *CH25H* genes were located in tandem on genomes [27]. The result of phylogenetic analysis showed that birds and mammals clustered in a distinct group in the phylogenetic tree of *CH25H* gene (Figure 3B). 

### 3.4. The Core Promoter of chCH25H

**Figure 4:**
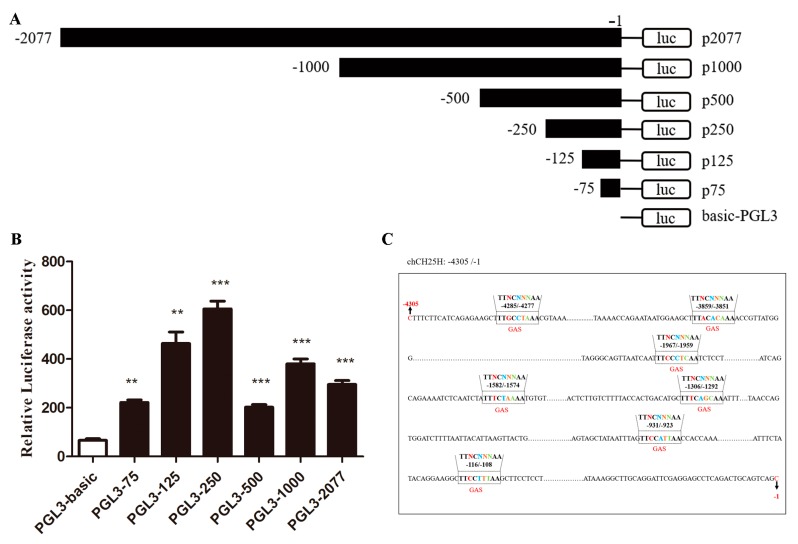
Analysis of the core promoter of *chCH25H*. (**A**) The first nucleotide of *chCH25H* coding region is numbered −1, and the different dual luciferase reporter vectors are named PGL3- p2077(−2077/−1), p1000 (−1000/−1), p500 (−500/−1), p250 (−250/−1), p125 (−125/−1) and p75 (−75/−1), respectively. (**B**) Dual luciferase assay showed that the inserted sequences of 75 bp, 125 bp, 250 bp, 500 bp, 1000 bp and 2077 bp in presumed promoter region all significantly increased the relative luciferase activity compared to the empty pGL3-basic vector in DF1 cells. (**C**) Screening of GAS and ISRE elements in the upstream region of *chCH25H*. ** *p* < 0.01; *** *p* < 0.001.

The result of dual luciferase assay showed that the inserted sequences of 75 bp, 125 bp, 250 bp, 500 bp, 1000 bp and 2077 bp in presumed promoter region all significantly increased the relative luciferase activity compared to the empty pGL3-Basic vector in DF1 cells (Figure 4B). These results indicated that the core promoter region of chicken *CH25H* was located within −75/−1 sequence. The upstream regions of *chCH25H* analysis showed that 7 GAS located in these regions, but no ISRE (Figure 4C).

### 3.5. Overexpression of chCH25H Inhibits ALV-J Replication

**Figure 5:**
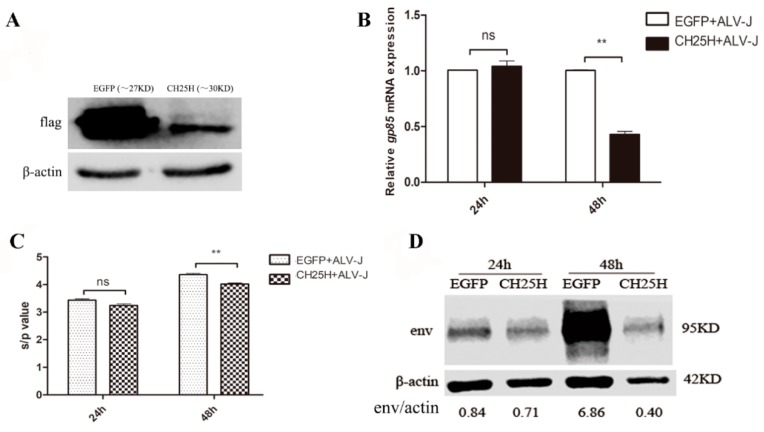
Overexpression of *chCH25H* inhibits ALV-J replication. (**A**) Exogenous *CH25H* and *EGFP* genes was expressed in DF1 cells at 24 h post transfection. (**B**) The expression of SCAU-HN06 *gp85* gene was detected by qPCR in DF1 cells at 24 and 48 hpi. (**C**) The expression of ALV-J p27 protein was detected by ELISA in DF1 cells at 24 and 48 hpi. (**D**) Expression levels of the ALV-J envelope protein in DF1 cells were detected by Western blotting using mouse antibody JE9. “CH25H” on the graph represents overexpression of *CH25H* in DF1 cells. “EGFP” represents control. ** *p* < 0.01.

In the overexpression studies described in Figure 5, DF1 cells were transfected with pflag-*CH25H* plasmid such that these cells highly expressed the *CH25H* gene. Figure 5A showed that exogenous *CH25H* and *EGFP* genes was expressed in DF1 cells at 24 h post transfection. The results of qPCR, ELISA, and western blot all demonstrated that overexpression of *chCH25H* could inhibit the expression of ALV-J p27 and envelope protein in DF1 cells at 48 hpi (Figure 5B–D). These results indicated that chicken *CH25H* significantly inhibited ALV-J replication.

### 3.6. Knockdown of Endogenous chCH25H Enhances ALV-J Replication

**Figure 6:**
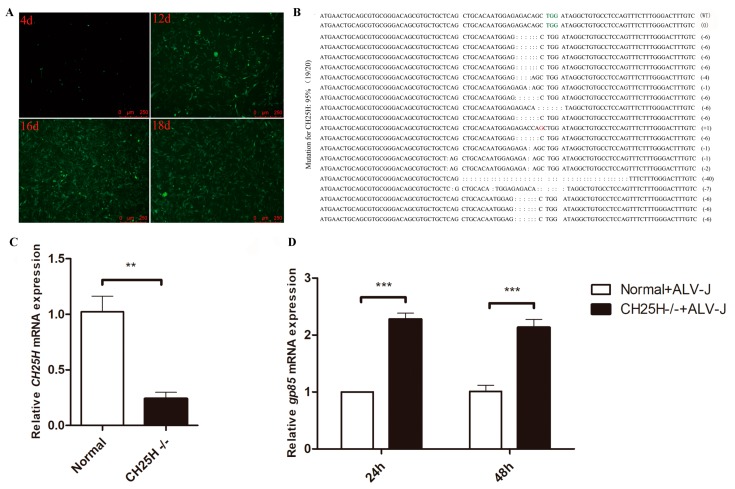
Knockdown of endogenous *chCH25H* enhanced ALV-J replication. (**A**) The green fluorescence was monitored in *chCH25H*-knockout cells from 4 to18 days. (**B**) The knockout efficiency of *chCH25H* in twenty monoclonal bacteria was determined by sequencing. (**C**) The expression level of *chCH25H* in *chCH25H*- knockout DF1 cells was detected by qPCR. Compared to normal DF1 cells, the *chCH25H* expression in *chCH25H-* knockout cells was significantly decreased. (**D**) qPCR results showed that knockdown of endogenous *chCH25H* could significantly increased the expression of ALV-J *gp85* gene. “CH25H−/−” represents *chCH25H-* knockout DF1 cells. “Normal” represents normal DF1 cells. ** *p* < 0.01; *** *p* < 0.001.

To further evaluate the antiviral effect of *chCH25H* on ALV-J replication, we applied CRISPR/Cas9 genome editing technology to knockdown the endogenous *chCH25H*. The DF1 cells transfected with Cas9/gRNA recombinant vector were screened by the 2.5 μg/mL puromycin for 18 days. The green fluorescent represented a proliferative process of the puromycin-selected cells from 4 to18 days (Figure 6A). Almost all knockout cells showed green fluorescence at 18 days (Figure 6A). The knockout efficiency in twenty monoclonal bacteria randomly selected for sequencing was 19/20 (95%) (Figure 6B). Accordingly, we judged that the knockout efficiency of the *chCH25H*-knockout cell line was 95%. Furthermore, compared to normal DF1 cells, the expression level of *chCH25H* in *chCH25H* knockout cells was significantly decreased (Figure 6C). qPCR results showed that the knockdown of endogenous *chCH25H* could significantly enhance ALV-J replication (Figure 6D).

### 3.7. chCH25H Produces A Soluble Antiviral Factor that Is Not Chicken Type I and II IFN

**Figure 7:**
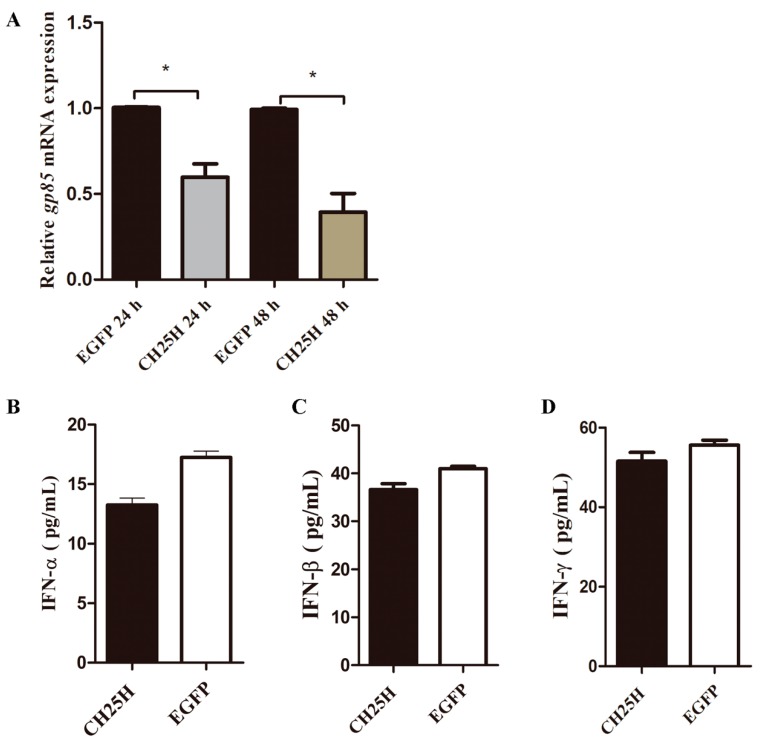
*chCH25H* produces a soluble anti-ALV-J factor that is not chicken type I and II IFN. (**A**) DF1 cells were pretreated with the cell supernatants collected from DF1 cells overexpressing *chCH25H* at 24 h post transfection. Compared to the cell supernatants collected from DF1 cells overexpressing *EGFP*, cell supernatants collected from DF1 cells overexpressing *chCH25H* could significantly inhibit the expression of ALV-J *gp85* gene at 24 and 48 hpi. (**B**–**D**) chIFN including IFN-α, IFN-β and IFN-γ were detected by ELISA in the cell supernatants collected from DF1 cells overexpressing *chCH25H*. Compared to control, no chIFN were significantly induced by overexpressing *chCH25H*. * *p* < 0.05.

To determine whether *chCH25H* produced a soluble antiviral factor, we detected whether medium collected from DF1 cells overexpressing *chCH25H* had antiviral activity. The results showed that cell supernatants collected from DF1 cells overexpressing *chCH25H* at 24 h post transfection could significantly inhibit the expression of ALV-J *gp85* gene at 24 and 48 hpi (Figure 7A). These results demonstrate that *chCH25H* produces a soluble antiviral factor in DF1 cells. 

Interferon induces many ISGs that positively feed back and amplify its production. To further verify whether the soluble antiviral factor produced by *chCH25H* is chIFN or not, we detect the chIFN by ELISA in the cell supernatants collected from DF1 cells overexpressing *chCH25H*. However, compared to control, no chIFN including IFN-α, IFN-β and IFN-γ were significantly induced by overexpressing *chCH25H* (Figure 7B–D). Taken together, *chCH25H* produces a soluble antiviral factor that is not type I and II chIFN.

### 3.8. 25HC, the Product of chCH25H, Has Anti-ALV-J Activity

**Figure 8:**
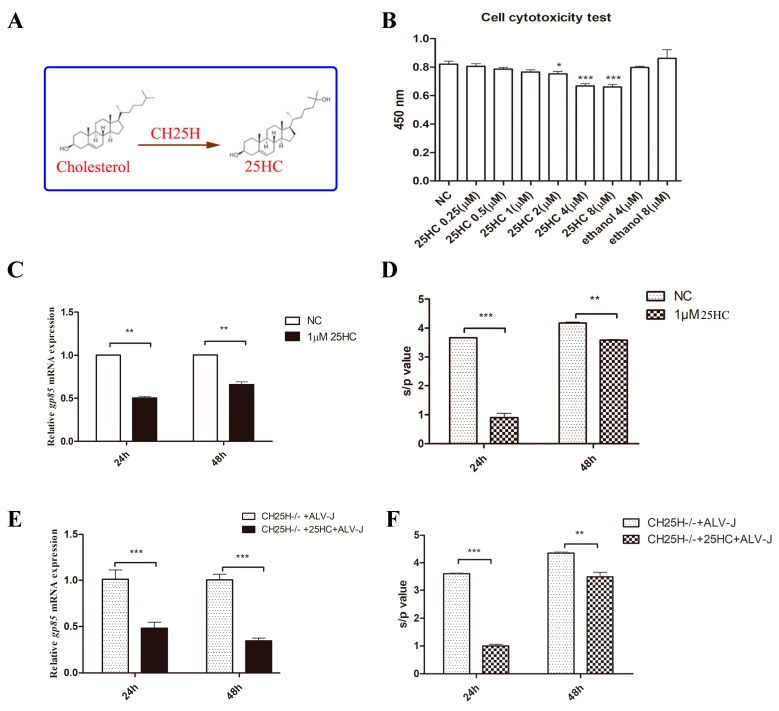
25HC, the product of *chCH25H*, inhibits ALV-J replication. (**A**) *CH25H* converts cholesterol to 25HC. (**B**) DF1 cells were seeded into a 96-well plate and treated with ethanol (4 and 8 μM) and different concentrations of 25HC (0.25, 0.5, 1, 2, 4, 8 μM) for 24 h. A cytotoxicity assay was performed with the Cell Counting Kit-8 dye kit. (**C**,**D**) DF1 cells were treated with 1μM 25HC for 12 h, followed by infection with ALV-J strain SCAU-HN06. The expression of ALV-J *gp85* mRNA and p27 protein was detected by qPCR (**C**) and ELISA (**D**) at 24 and 48 hpi. (**E**,**F**) *ChCH25H*- knockout DF1 cells were treated with 1 μM 25HC for 12 h, followed by infection with ALV-J strain SCAU-HN06. The expression of ALV-J *gp85* mRNA and p27 protein was detected by qPCR (**E**) and ELISA (**F**) at 24 and 48 hpi. DF1 cells and *chCH25H*-knockout DF1 cells pretreated with ethanol were used as control (NC). * *p* < 0.05; ** *p* < 0.01; *** *p* < 0.001.

*CH25H* catalyzes oxidation of cholesterol to 25HC (Figure 8A), a soluble oxysterol that acts as an antiviral factor to broadly inhibit the growth of enveloped viruses such as vesicular stomatitis virus (VSV), ebola virus (EBOV) and human immunodeficiency virus (HIV) [8]. The treatment of DF1 cells with different concentrations of 25HC for 24 h showed that 1 μM of 25HC is the highest concentration that is not toxic to cells (Figure 8B). The results of qPCR and p27 ELISA showed that the treatment of normal DF1 cells and *chCH25H* knockout DF1 cells with 1 μM of 25HC significantly inhibited the expression of ALV-J *gp85* mRNA and p27 protein at 24 and 48 hpi (Figure 8C–F). All of these results suggested that 25HC, a product of *CH25H*, could inhibt ALV-J replication in DF1 cells.

## 4. Discussion

ISGs have been found to be critical for controlling virus infections [3]. Their antiviral functions are gaining more and more attention in antiviral innate immune responses. Hundreds of human ISGs induced by IFN-α, IFN-β and IFN-γ have been identified with genome-wide transcriptional profiling [4,5,6]. Although IFN was discovered in chickens in 1957 [28], the first chicken IFN-induced gene was described in 1994 [29]. To date, few chicken ISGs such as *PKR* [30], *Mx* [31], *ZAP* [32], *Viperin* [33] and *IFIT5* [34] have been characterized, and their antiviral function have been explored. Importantly, more than hundred chicken ISGs have been identified by microarray and RNA-seq in primary chicken embryo fibroblast (CEF) [7]. However, there are still many chicken ISGs that need to be identified, and their antiviral mechanisms need to be elaborated.

In this study, 897 genes significantly up-regulated by chIFN-α in chicken PBMC were considered to be chicken type I ISGs. ISGs expression levels are often dependent on many factors such as treatment time, IFN and virus dose, and cell type [2]. Meantime, ISGs up-regulated by ALV-J infection were selected as potential anti-ALV-J ISG candidates. Accordingly, we systematically identified 152 potential anti-ALV-J chicken type I ISG candidates. As we all know that the interaction between virus and host is a very complicated process. Therefore, the potential anti-ALV-J gene identified by the method in this study does not represent a complete anti-ALV-J library. 

Furthermore, given that the broad antiviral effects of *CH25H* [8,10,17,18,19,20,21], *chCH25H*, an anti-ALV-J ISG candidate, was chosen to further explore its anti-ALV-J mechanism in DF1 cells. As an enzyme, *CH25H* catalyzes the cholesterol to form a soluble factor, 25HC [35]. Recent studies have showed that *CH25H* is broadly antiviral by producing 25HC to fight against several viruses such as mammalian reovirus [21], zika virus [17], hepatitis C virus [36], lassa virus [37] and porcine reproductive and respiratory syndrome virus [38]. The mechanism of antiviral function for 25HC is not completely understood. For enveloped viruses, 25HC suppressed viral replication by blocking membrane fusion between cells and viruses [8]. Whether *chCH25H* inhibit the replication of the notorious chicken retrovirus ALV-J or not? In our latest study, we found that overexpression of *chCH25H* in chicken primary macrophage could restrict ALV-J infection [39]. However, we did not explore the mechanism of *CH25H* against ALV-J infection in chicken macrophages.

*CH25H* is a conserved ISG in mammals, but lacks enough description in the chicken, especially its antiviral function. In the present study, we demonstrated that chIFN-α and ALV-J induced expression of *chCH25H* in chicken PBMC and DF1 cells (Figure 2). Thus, *chCH25H* is an interferon-stimulated gene in chicken. ISGs activation followed the binding of transcriptional complex and ISRE or GAS [40]. Of the hundreds of known ISGs in other species, some have only ISRE or only GAS elements in their upstream regions, whereas others have both elements [40]. In general, type I and type II IFNs induced the transcription of genes that have ISRE and GAS elements in their promoter, respectively [40]. However, upstream regions of *chCH25H* analysis showed that 8 GAS were located in these regions, but no ISRE (Figure 4C). Our result suggested that *chCH25H* not only can be induced by IFN-α, but also might be induced by IFN-γ. The upstream unknown region of the *chCH25H* still needs to be identified, with the hope to find ISRE elements in those regions.

Furthermore, we found that *chCH25H* and its product, 25HC, both inhibited ALV-J replication in DF1 cells. Previous studies have found that administration of 25HC could inhibit HIV-1 and Zika virus infection in animal models [8,17]. These results suggested that *CH25H* played an important physiologic role in antiviral defense. Given that no vaccine for ALV-J is currently available, *chCH25H* can be used as a new strategy to control ALV-J. 25HC could potentially be used as an anti-ALV-J therapeutic as it has in vitro activity against ALV-J. Although the antiviral mechanism of 25HC through affecting viral and cell membrane fusion has been widely found, other new antiviral mechanisms still need to be determined. However, the antiviral activity of 25HC against a number of viruses still highlights this oxysterol as a potential target for antiviral therapy. The further study on the underlying immunological mechanism of *chCH25H* and 25-HC in inhibiting ALV-J infection should be illuminated. Furthermore, transgenic chickens with overexpression of *chCH25H* in vivo deserve further research. These *CH25H*-transgenic chickens may have a broad-spectrum antiviral property.

## 5. Conclusions

In this study, we first identified 152 potential anti-ALV-J chicken type I ISGs. In addition, we further demonstrated that *chCH25H* was a chicken ISG and that it could inhibit ALV-J replication through 25HC in DF1 cells. These findings characterized a new chicken ISG and provided new insights into the control of ALV-J infection.

## Supplementary Materials

The following are available online at https://www.mdpi.com/1999-4915/11/6/498/s1.

## References

[1] Payne L.N., Nair V. (2012). The long view: 40 years of avian leukosis research. Avian Pathol..

[2] Schoggins J.W., Rice C.M. (2011). Interferon-stimulated genes and their antiviral effector functions. Curr. Opin. Virol..

[3] Schoggins J.W. (2014). Interferon-stimulated genes: Roles in viral pathogenesis. Curr. Opin. Virol..

[4] Liu S.Y., Sanchez D.J., Aliyari R., Lu S., Cheng G. (2012). Systematic identification of type I and type II interferon-induced antiviral factors. Proc. Natl. Acad. Sci. USA.

[5] Schoggins J.W., Wilson S.J., Panis M., Murphy M.Y., Jones C.T., Bieniasz P., Rice C.M. (2011). A diverse range of gene products are effectors of the type I interferon antiviral response. Nature.

[6] Zhang X., Yang W., Wang X., Zhang X., Tian H., Deng H., Zhang L., Gao G. (2018). Identification of new type I interferon-stimulated genes and investigation of their involvement in IFN-beta activation. Protein Cell.

[7] Giotis E.S., Robey R.C., Skinner N.G., Tomlinson C.D., Goodbourn S., Skinner M.A. (2016). Chicken interferome: Avian interferon-stimulated genes identified by microarray and RNA-seq of primary chick embryo fibroblasts treated with a chicken type I interferon (IFN-alpha). Vet. Res..

[8] Liu S.Y., Aliyari R., Chikere K., Li G., Marsden M.D., Smith J.K., Pernet O., Guo H., Nusbaum R., Zack J.A. (2013). Interferon-inducible cholesterol-25-hydroxylase broadly inhibits viral entry by production of 25-hydroxycholesterol. Immunity.

[9] Gold E.S., Diercks A.H., Podolsky I., Podyminogin R.L., Askovich P.S., Treuting P.M., Aderem A. (2014). 25-Hydroxycholesterol acts as an amplifier of inflammatory signaling. Proc. Natl. Acad. Sci. USA.

[10] Wu T., Ma F., Ma X., Jia W., Pan E., Cheng G., Chen L., Sun C. (2018). Regulating Innate and Adaptive Immunity for Controlling SIV Infection by 25-Hydroxycholesterol. Front. Immunol..

[11] Himly M., Foster D.N., Bottoli I., Iacovoni J.S., Vogt P.K. (1998). The DF-1 chicken fibroblast cell line: Transformation induced by diverse oncogenes and cell death resulting from infection by avian leukosis viruses. Virology.

[12] Zhang H., Guan Z.S., Guan S.H., Yang K., Pan Y., Wu Y.Y., Wang A.H., Sun B.B., Hou J., Mu X.X. (2016). The Study of Immune Response in PBMC of CHB Patients treated with IFN-alpha and 3-TC in vitro. Clin. Lab..

[13] Dai M., Feng M., Xie T., Li Y., Ruan Z., Shi M., Liao M., Zhang X. (2017). ALV-J infection induces chicken monocyte death accompanied with the production of IL-1beta and IL-18. Oncotarget.

[14] Langmead B., Salzberg S.L. (2012). Fast gapped-read alignment with Bowtie 2. Nat. Methods.

[15] Kim D., Pertea G., Trapnell C., Pimentel H., Kelley R., Salzberg S.L. (2013). TopHat2: Accurate alignment of transcriptomes in the presence of insertions, deletions and gene fusions. Genome Biol..

[16] Li B., Dewey C.N. (2011). RSEM: Accurate transcript quantification from RNA-Seq data with or without a reference genome. BMC Bioinform..

[17] Li C., Deng Y.Q., Wang S., Ma F., Aliyari R., Huang X.Y., Zhang N.N., Watanabe M., Dong H.L., Liu P. (2017). 25-Hydroxycholesterol Protects Host against Zika Virus Infection and Its Associated Microcephaly in a Mouse Model. Immunity.

[18] Ke W., Fang L., Jing H., Tao R., Wang T., Li Y., Long S., Wang D., Xiao S. (2017). Cholesterol 25-Hydroxylase Inhibits Porcine Reproductive and Respiratory Syndrome Virus Replication through Enzyme Activity-Dependent and -Independent Mechanisms. J. Virol..

[19] Zhang Y., Song Z., Wang M., Lan M., Zhang K., Jiang P., Li Y., Bai J., Wang X. (2019). Cholesterol 25-hydroxylase negatively regulates porcine intestinal coronavirus replication by the production of 25-hydroxycholesterol. Vet. Microbiol..

[20] Zhang Y., Wang L., Huang X., Wang S., Huang Y., Qin Q. (2019). Fish Cholesterol 25-Hydroxylase Inhibits Virus Replication via Regulating Interferon Immune Response or Affecting Virus Entry. Front. Immunol..

[21] Doms A., Sanabria T., Hansen J.N., Altan-Bonnet N., Holm G.H. (2018). 25-Hydroxycholesterol Production by the Cholesterol-25-Hydroxylase Interferon-Stimulated Gene Restricts Mammalian Reovirus Infection. J. Virol..

[22] Feng M., Tan Y., Dai M., Li Y., Xie T., Li H., Shi M., Zhang X. (2016). Endogenous Retrovirus ev21 Dose Not Recombine with ALV-J and Induces the Expression of ISGs in the Host. Front. Cell. Infect. Microbiol..

[23] Roll S., Hartle S., Lutteke T., Kaspers B., Hartle S. (2017). Tissue and time specific expression pattern of interferon regulated genes in the chicken. BMC Genom..

[24] Zhang Y., Wang Y., Zuo Q., Li D., Zhang W., Wang F., Ji Y., Jin J., Lu Z., Wang M. (2017). CRISPR/Cas9 mediated chicken Stra8 gene knockout and inhibition of male germ cell differentiation. PLoS ONE.

[25] Dai M., Feng M., Liu D., Cao W., Liao M. (2015). Development and application of SYBR Green I real-time PCR assay for the separate detection of subgroup J Avian leukosis virus and multiplex detection of avian leukosis virus subgroups A and B. Virol. J..

[26] Dai M., Feng M., Ye Y., Wu X., Liu D., Liao M., Cao W. (2016). Exogenous avian leukosis virus-induced activation of the ERK/AP1 pathway is required for virus replication and correlates with virus-induced tumorigenesis. Sci. Rep..

[27] Holmes R.S., Vandeberg J.L., Cox L.A. (2011). Genomics and proteomics of vertebrate cholesterol ester lipase (LIPA) and cholesterol 25-hydroxylase (CH25H). 3 Biotech..

[28] Isaacs A., Lindenmann J. (1957). Virus interference. I. The interferon. Proc. R. Soc. Lond. B. Biol. Sci..

[29] Sekellick M.J., Ferrandino A.F., Hopkins D.A., Marcus P.I. (1994). Chicken interferon gene: Cloning, expression, and analysis. J. Interferon Res..

[30] Ko J.H., Asano A., Kon Y., Watanabe T., Agui T. (2004). Characterization of the chicken PKR: Polymorphism of the gene and antiviral activity against vesicular stomatitis virus. Jpn. J. Vet. Res..

[31] Sasaki K., Yoneda A., Ninomiya A., Kawahara M., Watanabe T. (2013). Both antiviral activity and intracellular localization of chicken Mx protein depend on a polymorphism at amino acid position 631. Biochem. Biophys. Res. Commun..

[32] Goossens K.E., Karpala A.J., Ward A., Bean A.G. (2014). Characterisation of chicken ZAP. Dev. Comp. Immunol..

[33] Goossens K.E., Karpala A.J., Rohringer A., Ward A., Bean A.G. (2015). Characterisation of chicken viperin. Mol. Immunol..

[34] Santhakumar D., Rohaim M., Hussein H.A., Hawes P., Ferreira H.L., Behboudi S., Iqbal M., Nair V., Arns C.W., Munir M. (2018). Chicken Interferon-induced Protein with Tetratricopeptide Repeats 5 Antagonizes Replication of RNA Viruses. Sci Rep..

[35] Lund E.G., Kerr T.A., Sakai J., Li W.P., Russell D.W. (1998). cDNA cloning of mouse and human cholesterol 25-hydroxylases, polytopic membrane proteins that synthesize a potent oxysterol regulator of lipid metabolism. J. Biol. Chem..

[36] Xiang Y., Tang J.J., Tao W., Cao X., Song B.L., Zhong J. (2015). Identification of Cholesterol 25-Hydroxylase as a Novel Host Restriction Factor and a Part of the Primary Innate Immune Responses against Hepatitis C Virus Infection. J. Virol..

[37] Shrivastava-Ranjan P., Bergeron E., Chakrabarti A.K., Albarino C.G., Flint M., Nichol S.T., Spiropoulou C.F. (2016). 25-Hydroxycholesterol Inhibition of Lassa Virus Infection through Aberrant GP1 Glycosylation. mBio.

[38] Song Z., Zhang Q., Liu X., Bai J., Zhao Y., Wang X., Jiang P. (2017). Cholesterol 25-hydroxylase is an interferon-inducible factor that protects against porcine reproductive and respiratory syndrome virus infection. Vet. Microbiol..

[39] Feng M., Xie T., Li Y., Zhang N., Lu Q., Zhou Y., Shi M., Sun J., Zhang X. (2019). A balanced game: Chicken macrophage response to ALV-J infection. Vet. Res..

[40] Platanias L.C. (2005). Mechanisms of type-I- and type-II-interferon-mediated signalling. Nat. Rev. Immunol..

